# Echocardiographic assessment of cardiac involvement in pediatric COVID-19 patients: a cross-sectional study

**DOI:** 10.1186/s12887-026-07039-3

**Published:** 2026-05-25

**Authors:** Mohammad Reza Khalilian, Aliahmad Goudarzi, Abdollah Karimi, Alireza Fahimzad, Kourosh Vahidshahi, Fariba Alaei, Tahmineh Tahouri

**Affiliations:** 1 Department of Pediatrics, School of Medicine, Shahid Beheshti University of Medical Sciences, Tehran, Iran; 2 Department of Pediatrics, Shahid Beheshti University of Medical Science, Tehran, Iran; 3 Pediatric Infections Research Center, Research Institute for Children’s Health, Shahid Beheshti University of Medical Sciences, Tehran, Iran; 4 Pediatric Infectious Research Center, Research Institute for Children’s Health, Shahid Beheshti University of Medical Sciences, Tehran, Iran; 5 Pediatric Cardiology Ward, Modarres Teaching Hospital, Shahid Beheshti University of Medical Sciences, Tehran, Iran; 6 Department of Pediatric Cardiology, Mofid Children’s Hospital, Shahid Beheshti University of Medical Science, Tehran, Iran; 7 Cardiovascular Research Center, Rajaie Cardiovascular Institute, Valiasr Ave., Niayesh Intersection, Tehran, 1995614331 Iran

**Keywords:** COVID-19, Multisystem Inflammatory Syndrome in Children, Pediatrics, Echocardiography, Ventricular Dysfunction, Myocarditis, Child, Cardiac Imaging Techniques

## Abstract

**Background:**

Cardiac involvement has emerged as a significant complication in children with COVID-19, particularly in those with multisystem inflammatory syndrome in children (MIS-C). However, systematic echocardiographic data remain limited, especially from the Middle East.

**Methods:**

In this descriptive cross-sectional study, 433 children with confirmed COVID-19 admitted to a tertiary hospital in Tehran were evaluated. Transthoracic echocardiography was performed in all patients during hospitalization. Cardiac function, valvular regurgitation, pericardial and pleural effusion, and coronary artery dimensions (Z-score–based) were recorded. Findings were compared between MIS-C and non–MIS-C patients.

**Results:**

Among 433 patients (mean age 4.56 years; 54% male), 270 (62.5%) were diagnosed with MIS-C. The most common echocardiographic abnormalities were mitral regurgitation (19.4%) and pericardial effusion (18.5%). Coronary abnormalities (1.4%) and LV systolic dysfunction (4.8%) were uncommon. MIS-C patients had significantly higher rates of pericardial effusion (23% vs. 11%, *p* = 0.002). Pericardial effusion was the only variable significantly associated with ICU admission (*p* = 0.002). Pleural effusion was observed only in non-MIS-C patients (3.1%, *p* = 0.003).

**Conclusions:**

Echocardiographic abnormalities are common in children hospitalized with COVID-19, particularly those with MIS-C. Pericardial effusion may serve as an early marker of severity and guide triage. Routine cardiac imaging is recommended in hospitalized children with inflammatory symptoms.

## Introduction

Since its emergence in Wuhan, China in December 2019, coronavirus disease 2019 (COVID-19), caused by the novel severe acute respiratory syndrome coronavirus 2 (SARS-CoV-2), has rapidly evolved into a global pandemic. The World Health Organization (WHO) officially declared the outbreak a pandemic in March 2020 [1]. Initially, it was believed that children were relatively spared from the severe consequences of COVID-19, which predominantly affected adults—particularly the elderly and those with underlying comorbidities [2].

However, as the pandemic progressed, a rare but serious pediatric inflammatory condition known as Multisystem Inflammatory Syndrome in Children (MIS-C) began to emerge. This syndrome, associated with recent SARS-CoV-2 infection or exposure, presents with persistent fever, laboratory evidence of inflammation, and multisystem organ involvement. The United States Centers for Disease Control and Prevention (CDC) have established diagnostic criteria for MIS-C, including fever > 38 °C for at least 24 h, elevated inflammatory markers (e.g., CRP, ESR, ferritin, D-dimer, IL-6), and evidence of dysfunction in two or more organ systems (e.g., cardiac, gastrointestinal, dermatologic, neurologic), in the absence of an alternative plausible diagnosis [3].

Although respiratory symptoms remain the most common manifestation of COVID-19 in children, increasing evidence has highlighted the occurrence of multi-organ involvement, including the cardiovascular system [4]. Cardiac complications associated with MIS-C are particularly concerning due to their severity and potential for long-term consequences. These complications can range from mild cardiac enzyme elevation to life-threatening conditions such as myocarditis, arrhythmias, pericardial effusion, coronary artery aneurysms, and cardiogenic shock [5]. Some patients exhibit dramatic and acute cardiac dysfunction, while others may have subclinical findings detectable only through imaging.

Given the diversity and severity of cardiac presentations, comprehensive cardiac evaluation—including laboratory markers (e.g., troponin, BNP), electrocardiography (ECG), transthoracic echocardiography, and, when indicated, cardiac magnetic resonance imaging (CMR)—is essential in children suspected of MIS-C. However, few studies have systematically assessed the spectrum of cardiac involvement in hospitalized children with COVID-19, particularly in the Middle East.

This study aims to describe and compare the echocardiographic findings of children with confirmed COVID-19 infection, with and without a final diagnosis of MIS-C, admitted to a tertiary pediatric center in Tehran, Iran. The results are intended to provide insight into the prevalence and patterns of cardiac abnormalities in this population and to support early cardiac assessment in similar clinical settings.

## Material and methods

### Study population

This descriptive, cross-sectional, single-center study, included 433 patients under 18 years of age who were hospitalized at Mofid Children’s Hospital, Tehran, Iran, between February 1, 2020, and September 31, 2021, with a confirmed diagnosis of COVID-19.

Inclusion criteria were: age under 18 years, hospitalization with laboratory or epidemiological evidence of current or recent SARS-CoV-2 infection, including positive RT-PCR, serology, antigen testing, or documented COVID-19 exposure within the 4 weeks prior to symptom onset; and written informed consent obtained from a parent or legal guardian. Exclusion criteria comprise: known pre-existing congenital or acquired heart disease, chronic pulmonary or systemic disease. Patients were enrolled consecutively upon admission to ensure unbiased sampling. Assent from older children was not obtained due to institutional policy and the acute clinical circumstances surrounding patient admission.

No additional financial burden was imposed on the patients during this study. All patient information was treated as confidential, and the researcher adhered to the principles outlined in the Helsinki Convention and approved by the Ethical Committee of Shahid Beheshti University of Medical Science.

The sample entry process followed a gradual approach, documenting demographic and clinical details through a questionnaire. A comprehensive record, encompassing detailed history and physical examination data (age, gender, weight, height) and echocardiography findings was maintained.

### Echocardiographic evaluation

Transthoracic echocardiography was performed for all enrolled patients during hospitalization, regardless of clinical presentation. Examinations were conducted by a single experienced pediatric cardiologist using a Samsung HS70 ultrasound system (Samsung, Republic of Korea), following standard pediatric echocardiographic protocols.

Standard imaging views included parasternal long-axis and short-axis, apical four-chamber, subcostal, and suprasternal notch views. Quantitative assessment was used for ventricular function: left ventricular ejection fraction (LVEF) was calculated using M-mode imaging in the parasternal long-axis view, and right ventricular systolic function was assessed using tricuspid annular plane systolic excursion (TAPSE).

Left ventricular diastolic function was evaluated using mitral inflow Doppler and tissue Doppler imaging at the mitral annulus. Right ventricular diastolic function was assessed using tricuspid inflow Doppler and annular TDI. Pulmonary artery systolic pressure was estimated from the peak tricuspid regurgitation velocity using the modified Bernoulli equation.

Valvular abnormalities (mitral and aortic regurgitation), pericardial and pleural effusions, and intracardiac thrombi were also documented. Mitral and aortic valve regurgitation were assessed using color Doppler imaging. Valvular insufficiencies such as mitral and aortic regurgitation were noted but formal grading of severity (e.g., mild, moderate, severe) was not performed. Therefore, some mild cases may represent physiological findings commonly observed in healthy children. Mitral regurgitation (MR) was defined as pathological if the regurgitant jet extended more than 1 cm into the left atrium in apical 4-chamber view, was holosystolic in duration, or was associated with structural mitral valve abnormalities (e.g., prolapse, cleft, or thickening). Cases with faint or trivial jets limited to early systole, without structural valve changes, were considered physiological and excluded from the pathological MR category.

Coronary artery dimensions were measured at the proximal segments of the left main coronary artery (LMCA), left anterior descending artery (LAD), and right coronary artery (RCA). Z-scores were calculated based on body surface area. A Z-score ≥ 2 was considered dilated, and ≥ 2.5 was classified as aneurysmal.

All echocardiographic measurements and definitions were performed in accordance with the Guidelines for Performing a Comprehensive Pediatric Transthoracic Echocardiogram and the recommendations of the American Society of Echocardiography for quantification methods in pediatric echocardiography [6, 7]. Coronary artery Z-scores were calculated according to the equations proposed by Dallaire and Dahdah, and pathological mitral regurgitation was defined based on published pediatric normative data [8].

**Table:** Diagnosis of MIS-C was made according to the United States Center for Disease Control and Prevention (CDC) table:

1. Person less than 21 years of age presenting with fever^1^, laboratory evidence of inflammation^2^, and evidence of severe clinical disease requiring hospitalization, with multi-system (≥ 2 organs) involvement (cardiac, renal, respiratory, hematologic, Gastrointestinal, dermatologic, or neurologic); AND
2. No alternative possible diagnosis; AND
3. Positive for current or recent SARS-COV-2 infection by RT-PCR, serology, or antigen test; or COVID-19 exposure within the 4-week prior to the onset of symptoms

^1^Fever > 38 ° C for ≥ 24 h, or report of subjective fever lasting ≥ 24 h

^2^including, but not limited to, 1 or more of the following: an elevated C-reactive protein (CRP), erythrocyte sedimentation rate (ESR), fibrinogen, procalcitonin, D-dimer, ferritin, lactic acid dehydrogenase (LDH), or interleukin 6 (IL-6), elevated neutrophils, reduced lymphocytes, and low albumin

### Statistical analysis

All statistical analyses were conducted using SPSS for Windows, version 22.0 (SPSS Inc., Chicago, IL, USA). Continuous variables were expressed as mean ± standard deviation (SD), and categorical variables as frequencies and percentages. The Kolmogorov–Smirnov test was used to assess the normality of continuous variables. Group comparisons for categorical variables were performed using the chi-square test or Fisher’s exact test, as appropriate. Continuous variables were compared using the Student’s t-test. A *p*-value < 0.05 was considered statistically significant. Patients with incomplete echocardiographic data were excluded from the final analysis. No imputation for missing data was performed. Multivariable analysis to adjust for potential confounders (such as age, sex, or ICU admission) was not performed. Therefore, observed associations should be interpreted with caution, as confounding may influence the results.

## Results

### Background characteristics of patients

A total of 433 children with confirmed COVID-19 infection were included in the study. Of these, 234 (54%) were male and 199 (46%) were female. The mean age of the study population was 4.56 ± 4.33 years. Among all patients, 408 (94.2%) were admitted to the pediatric ward and 25 (5.8%) required intensive care unit (ICU) admission at the time of echocardiographic evaluation. Ten patients (2.3%) died during hospitalization. The final diagnosis for 163 patients was COVID-19, and 270 patients were diagnosed with MIS-C (Table 1).

Baseline demographic and clinical characteristics are presented in Table 1. The mean age was 4.13 ± 4.11 years in the MIS-C group and 5.26 ± 4.59 years in the COVID-19 group (*p* = 0.04). There was no statistically significant difference between the two groups in terms of sex distribution, ICU admission, in-hospital mortality, or length of hospital stay (all *p* > 0.05).

**Table 1:** Baseline demographic and clinical characteristics of patients at admission, stratified by final diagnosis (MIS-C vs. COVID-19)

Variable	Total	Groups Based on Final Diagnosis		*p* value
		MIS-C *n* = 270	COVID-19 *n* = 163	
Age (years), Mean ± SD	4.56 ± 4.33	4.13 ± 4.11	5.26 ± 4.59	0.04
Gender, *n* (%)				0.93
Male	234 (54%)	145 (53.7%)	89 (54.6%)	
Female	199 (46%)	125 (46.3%)	74 (45.4%)	
Death, *n* (%)	10 (2.3%)	4 (1.5%)	6 (3.68%)	0.25
ICU admission, *n *(%)	25 (5.8%)	13 (4.8%)	12 (7.36%)	0.37
Hospital stays (days), Mean ± SD	3.79 ± 2.81	3.68 ± 2.51	3.98 ± 3.24	0.41

Baseline demographic and clinical characteristics of patients at admission, stratified by final diagnosis (MIS-C vs. COVID-19). Data are presented as mean ± standard deviation for continuous variables and number (percentage) for categorical variables. Comparisons between MIS-C and COVID-19 groups were performed using independent t-test for continuous variables and chi-square test for categorical variables. MIS-C, Multisystem inflammatory syndrome in children

### Echocardiographic findings in the total study population

**Fig. 1:**
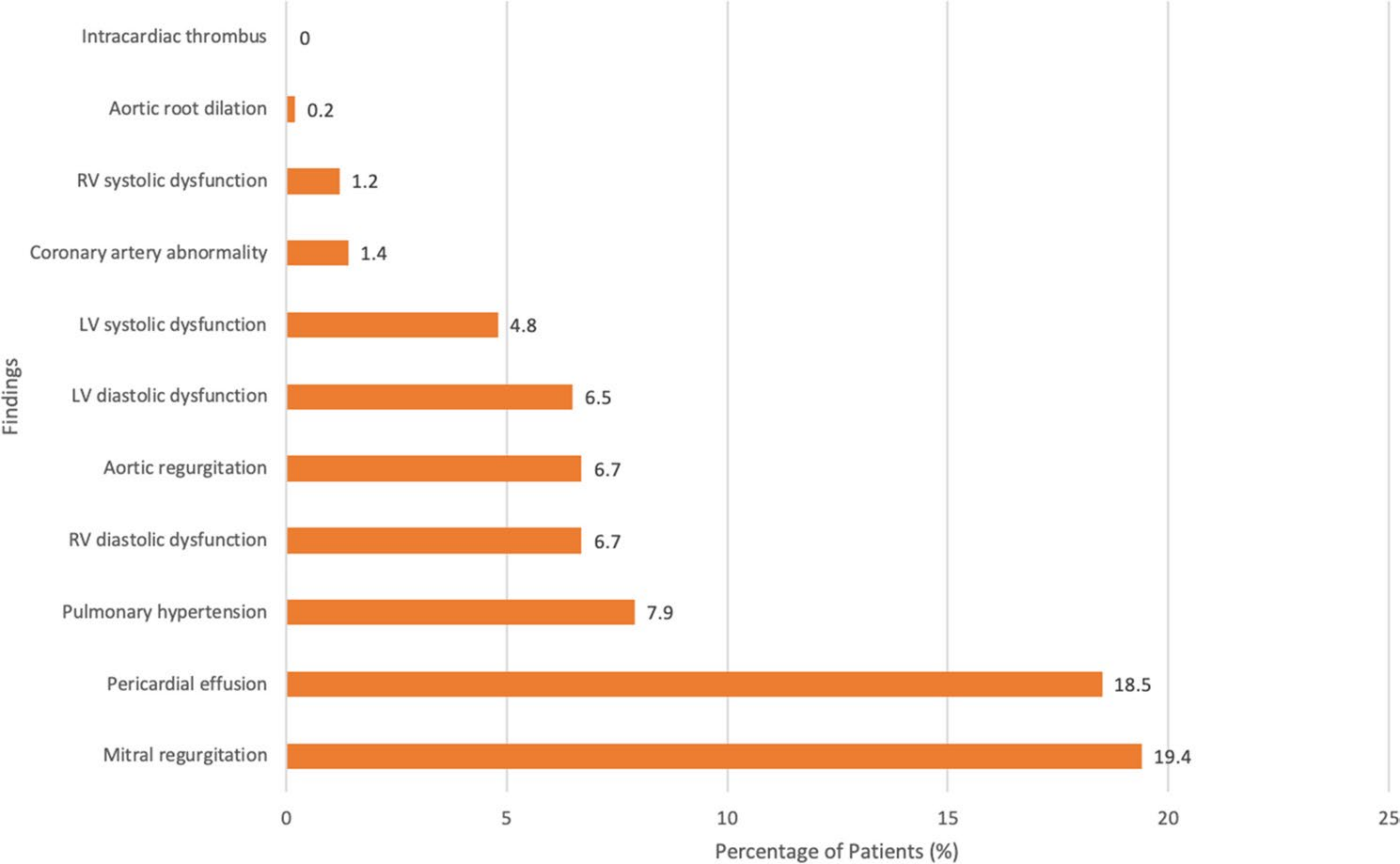
Frequency of Echocardiographic Abnormalities in All Patients

The overall pattern of echocardiographic findings in the study population (*n* = 433) is summarized in Fig. 1. Mitral regurgitation was observed in 19.4% of patients and pericardial effusion in 18.5%. Pulmonary hypertension was present in 7.9% of patients. Left ventricular systolic dysfunction was observed in 4.8% and left ventricular diastolic dysfunction in 5.5% of patients. Right ventricular diastolic dysfunction was present in 6.7%, while right ventricular systolic dysfunction was observed in 1.2%. Aortic regurgitation was present in 6.7% of patients. Aortic root dilation was identified in 1 patient (0.2%). Coronary artery abnormalities were observed in 1.4% of patients. No intracardiac thrombi were detected in any patient.

### Comparison of echocardiographic findings between MIS-C and COVID-19 patients

Table 2 summarizes the echocardiographic findings in patients with a final diagnosis of COVID-19 versus MIS-C.

**Table 2:** Comparison of Echocardiographic Findings in patients with COVID-19 and MIS-C

Echocardiographic Parameter	Total (*N* = 433)	COVID-19 (*n* = 163)	MIS-C (*n* = 270)	*p*-value
LV systolic function abnormal	21 (4.8%)	9 (5.5%)	12 (4.4%)	0.68
LV diastolic function abnormal	28 (6.5%)	13 (8.0%)	15 (5.6%)	0.31
RV systolic function abnormal	5 (1.2%)	4 (2.5%)	1 (0.4%)	0.07
RV diastolic function abnormal	29 (6.7%)	12 (7.4%)	17 (6.3%)	0.65
Mitral regurgitation (pathological)*	84 (19.4%)	27 (16.6%)	57 (21.1%)	0.24
Aortic regurgitation	29 (6.7%)	14 (8.6%)	15 (5.6%)	0.18
Pericardial effusion	80 (18.5%)	18 (11.0%)	62 (23.0%)	0.002
Pleural effusion	5 (1.2%)	5 (3.1%)	0 (0%)	0.003
Coronary artery involvement	6 (1.4%)	1 (0.6%)	5 (1.9%)	0.26
Pulmonary hypertension	34 (7.9%)	17 (10.4%)	17 (6.3%)	0.09
Aortic root dilation	1 (0.2%)	0 (0%)	1 (0.4%)	–
Intracardiac thrombus	0 (0%)	0 (0%)	0 (0%)	–

*Pathological mitral regurgitation was defined as a regurgitant jet>1 cm in length, holosystolic duration, or associated with mitral valve structural abnormalities

*Data are presented as number of patients (percentage)

*LV left ventricle, *RV *right ventricle, *MIS-C* multisystem inflammatory syndrome in children

Left ventricular systolic function was preserved in 94.5% of COVID patients and 95.6% of MIS-C patients. Diastolic function of the left ventricle was normal in 92% and 94.4% of COVID-19 and MIS-C patients, respectively. Right ventricular systolic and diastolic function were preserved in both groups. Pathological mitral valve regurgitation was observed in 21.1% of MIS-C patients and 16.6% of COVID-19 patients, although this difference was not statistically significant (*p* = 0.24). Pericardial effusion was observed in 23% of MIS-C patients and 11% of COVID-19 patients (*p* = 0.002).

Aortic valve regurgitation and coronary abnormalities were observed in both groups, with no statistically significant differences. Notably, pleural effusion was only observed in patients with COVID-19 (3.1%) with none in the MIS-C group (*p* = 0.003). Pulmonary hypertension was observed in 10.4% of COVID-19 patients and 6.3% of MIS-C patients (*p* = 0.09).

### Echocardiographic findings in ICU vs. Non-ICU patients

A subgroup analysis was performed to compare echocardiographic findings between patients admitted to the intensive care unit (ICU) (*n* = 25) and those admitted to the general pediatric ward (*n* = 408). Among the MIS-C patients, pericardial effusion was significantly more common in those requiring ICU care (*p* = 0.002). No other echocardiographic variables, including systolic or diastolic dysfunction, valve regurgitation, or coronary abnormalities, showed statistically significant difference between ICU and non-ICU patients (Table 3).

**Table 3:** Comparison of Echocardiographic Findings Between ICU and Non-ICU Patients

Echocardiographic Finding	ICU (*n* = 25)	Ward (*n* = 408)	*p*-value
Mitral Regurgitation	6 (24.0%)	78 (19.1%)	0.54
Aortic Regurgitation	2 (8.0%)	27 (6.6%)	0.68
Pericardial Effusion	10 (40.0%)	70 (17.2%)	0.002
Pleural Effusion	1 (4.0%)	4 (1.0%)	0.31
LV Systolic Dysfunction	2 (8.0%)	23 (5.6%)	0.64
LV Diastolic Dysfunction	1 (4.0%)	27 (6.6%)	0.55
RV Systolic Dysfunction	1 (4.0%)	4 (1.0%)	0.31
RV Diastolic Dysfunction	2 (8.0%)	27 (6.6%)	0.68
Coronary Artery Abnormalities	1 (4.0%)	5 (1.2%)	0.29
Pulmonary Hypertension	3 (12.0%)	31 (7.6%)	0.43
Aortic Root Dilatation	0 (0.0%)	1 (0.2%)	0.81
Intracardiac Thrombus	0 (0.0%)	0 (0.0%)	–

*Data are presented as number of patients (percentage). Pathological mitral and aortic regurgitation were defined as described in the Methods section. *ICU*  intensive care unit, *LV*  left ventricle, *RV*  right ventricle

## Discussion

In this large single-center study of 433 children hospitalized with COVID-19, we evaluated the prevalence and patterns of echocardiographic abnormalities, with particular attention to differences between patients with multisystem inflammatory syndrome in children (MIS-C) and those with acute COVID-19 alone. Our findings underscore that cardiac involvement is not uncommon, even in children without overt cardiac symptoms. The most frequent abnormalities identified were mitral regurgitation (19.4%) and pericardial effusion (18.5%). These findings were more prominent in patients with MIS-C and in those requiring intensive care, supporting the concept that cardiac manifestations are more common and more severe in hyperinflammatory COVID-19 phenotypes.

Our results are in line with the growing body of literature describing cardiac involvement in MIS-C. Studies by Belhadjer et al. [9], Valverde et al. [10], and Matsubara et al. [11] all report high rates of cardiac abnormalities in MIS-C, particularly systolic dysfunction, pericardial effusion, and valvular insufficiency. In our study, the overall prevalence of left ventricular systolic dysfunction was relatively low (5.8%), and coronary artery abnormalities were rare (1.2%). This contrasts with reports from Western cohorts, where LV dysfunction has been reported in up to 45% of MIS-C cases, and coronary artery dilatation in 8–20%. For example, Belhadjer et al. described acute LV dysfunction in 45% of children with MIS-C presenting with cardiogenic shock, with most requiring inotropic support [9]. Similarly, Valverde et al. found that 39% of MIS-C patients had impaired LVEF on initial echocardiography, many of whom recovered within days after treatment with IVIG and corticosteroids [10]. A large multi-center cohort from the United States reported LV dysfunction in over one-third of MIS-C patients requiring ICU admission [11]. Several factors may explain these differences. First, echocardiography in our patients was typically performed early in the disease course, which may have led to underestimation of transient or evolving abnormalities. Second, our study included a large proportion of children with non-MIS-C COVID-19, and only a minority required ICU care, suggesting overall milder disease. Third, differences in host genetics, viral variants, or healthcare system factors may contribute to variation across geographic regions.

The relatively high prevalence of mitral regurgitation (MR) in our study identified in nearly one-fifth (19.4%) of patients, warrants careful interpretation. In the context of MIS-C, MR may reflect transient myocardial inflammation, papillary muscle dysfunction, or annular dilation secondary to volume overload or ventricular dilation during the acute inflammatory phase. Several studies have reported MR as a frequent echocardiographic finding in MIS-C. For example, Matsubara et al. found MR in over 30% of MIS-C patients, often in association with left ventricular systolic dysfunction or impaired myocardial strain [8]. Similarly, Capone et al. and Valverde et al. reported MR in 20–45% of cases, with many resolving over time following treatment with immunomodulators or inotropes [12].

It is also important to note that trace or mild MR is common in healthy children, particularly neonates and young infants, due to physiological factors such as a thin mitral annulus, high heart rate, and incomplete leaflet coaptation. Several pediatric echocardiographic studies have shown that up to 16% of healthy children may exhibit physiological (non-pathologic) MR detectable on color Doppler imaging [13, 14].

In our study, the absence of formal MR grading criteria, such as vena contracta width, regurgitant volume, or jet density, limits our ability to differentiate between physiological and pathological MR. Therefore, it is likely that the observed MR rate reflects a mixture of benign and disease-related regurgitation. Nonetheless, the trend toward a higher frequency of MR in MIS-C compared to non-MIS-C COVID-19 patients suggests a possible role of ventricular dilation or valvulitis, both of which have been described in histopathological studies of MIS-C-related myocarditis [15].

Coronary artery abnormalities were identified in only 1.2% of patients in our cohort, despite the routine use of Z-score–based assessment during echocardiographic evaluation. This is considerably lower than the 6–20% prevalence reported in MIS-C cohorts from Europe, North America, and Asia. For example, the international registry by Valverde et al. found coronary dilatation or aneurysms in 24% of children with MIS-C [10], and Whittaker et al. reported similar coronary artery aneurysm in 14% of 58 patients with MIS-C in a UK-based cohort [16] and Lee et al. observed coronary involvement in 16% of children with MIS-C [17]. There are several possible explanations for this discrepancy. One key factor is the timing of echocardiography. In our study, echocardiograms were typically performed early in the hospitalization, often within the first 24–48 h of diagnosis. In contrast, many published cohorts include patients imaged during the subacute or convalescent phases, when coronary dilatation may be more apparent, particularly in children with persistent inflammation or delayed treatment. Indeed, delayed coronary involvement has been observed in MIS-C patients even after clinical stabilization, often emerging 5–10 days after admission [12, 18].

Another possibility is regional variation in immune response, viral variant, or host susceptibility. Coronary artery dilatation in MIS-C appears to have immunopathologic overlap with Kawasaki disease (KD), including endothelial dysfunction and systemic vasculitis. However, MIS-C often presents in older children and with more prominent myocardial dysfunction than KD, and the overall incidence of aneurysms seems lower than in classic KD cohorts [19]. The low prevalence of coronary changes in our patients may therefore reflect differences in genetic background, disease severity, timing of intervention, or even differences in diagnostic thresholds across regions.

Importantly, most coronary artery abnormalities reported in MIS-C are mild and transient. Follow-up studies show that 95–100% of coronary dilatations regress within 3 months, particularly in patients treated early with IVIG and corticosteroids [11, 12]. Thus, our findings may underestimate the true prevalence of coronary involvement, and highlight the importance of serial echocardiographic monitoring, especially in patients with persistent fever, elevated inflammatory markers, or incomplete response to treatment.

One critical limitation in relying solely on LVEF to assess myocardial involvement is its lack of sensitivity for detecting subclinical dysfunction. Several studies have demonstrated that global longitudinal strain (GLS) and myocardial deformation imaging via speckle-tracking echocardiography can detect subclinical myocardial injury in MIS-C patients with normal ejection fraction. Recent studies by Minocha et al. and Ahmed et al. have demonstrated impaired global longitudinal strain in a substantial proportion of children with MIS-C, even in the presence of preserved left ventricular ejection fraction [20, 21]. In our setting, speckle-tracking echocardiography and cardiac MRI were not routinely available. Therefore, the true burden of subclinical myocardial involvement may have been underestimated. Cardiac MRI, in particular, has revealed myocardial edema, late gadolinium enhancement, and ongoing myocardial inflammation in up to 50–75% of MIS-C patients, even weeks after discharge [22, 23].

These findings underscore the importance of utilizing advanced imaging modalities when available and highlight the need for longitudinal follow-up in MIS-C patients, even those with preserved systolic function on standard echocardiography. Without these tools, reliance on LVEF alone may lead to underrecognition of cardiac involvement, particularly in patients with milder or resolving symptoms.

In our study, pericardial effusion was the only echocardiographic finding that demonstrated a statistically significant association with ICU-level care, reinforcing its potential as a surrogate marker for disease severity. This observation aligns with multiple studies reporting high rates of pericardial effusion in MIS-C. for example, Valverde et al. documented pericardial effusion in 28% of children with MIS-C, often co-occurring with ventricular dysfunction or elevated inflammatory markers [10]. In a U.S.-based study, Matsubara et al. observed pericardial effusion in 32% of children with MIS-C, and noted a correlation between its presence and the need for inotropes and ICU admission [11]. Importantly, pericardial effusion is readily detected by focused point-of-care ultrasound (POCUS) or formal transthoracic echocardiography, making it an accessible and reproducible imaging target in both emergency and inpatient settings [24]. Its presence, particularly in a child with fever, shock, or elevated inflammatory markers, may warrant intensified cardiac monitoring, early escalation of care, or admission to higher acuity units. Moreover, as it does not require contrast or advanced imaging modalities, pericardial effusion can be tracked longitudinally in resource-limited settings.

Given these factors, we propose that routine screening for pericardial effusion should be incorporated into the initial echocardiographic evaluation of pediatric patients with COVID-19 and suspected MIS-C. Further research is needed to clarify whether the volume or rate of change of pericardial fluid correlates more closely with clinical deterioration or myocardial injury.

In contrast to patients with MIS-C, children with acute COVID-19 without MIS-C in our cohort demonstrated largely preserved cardiac function, with only a small proportion showing systolic or diastolic ventricular abnormalities on echocardiography. Significant structural cardiac involvement, including coronary artery abnormalities and pericardial effusion, was uncommon in this group. These findings suggest that, in the absence of a multisystem hyperinflammatory response, cardiac manifestations in pediatric COVID-19 are generally mild and may reflect nonspecific or transient changes rather than overt myocardial injury. Notably, pleural effusion was observed exclusively among non–MIS-C patients, supporting a more pulmonary-predominant pattern of disease in this subgroup compared with the cardiac-centered phenotype observed in MIS-C.

Several studies have documented this distinction. In a large review of MIS-C cases by Feldstein et al. pulmonary manifestations were far less common than gastrointestinal or cardiovascular symptoms, and chest radiography often lacked features of pneumonia or significant effusion [25]. Similarly, Radia et al.’s systematic review showed that pulmonary findings were present in only ~ 10% of MIS-C cases, often as incidental findings or reactive changes [26]. In contrast, pleural effusion in pediatric COVID-19 (non-MIS-C) has been linked to pneumonic consolidation, ARDS, or high inflammatory burden, as noted in observational cohorts from Wuhan and New York [27, 28].

It is also worth noting that pleural effusion in children may arise from non-cardiac causes, including renal dysfunction, hypoalbuminemia, and systemic capillary leak, particularly in the setting of prolonged fever or cytokine-driven inflammation. In MIS-C, the absence of pleural effusion may reflect a more cardiocentric pattern of systemic illness, characterized by myocardial injury, coronary involvement, and vasculitis-like syndromes, rather than alveolar-capillary damage.

Our findings reinforce the distinct pathophysiological pathways underlying MIS-C and acute COVID-19 in children. While the former appears to be a post-infectious hyperinflammatory syndrome with prominent cardiovascular and gastrointestinal involvement, the latter often retains viral tropism for the lungs, particularly in patients with respiratory symptoms at admission.

Future studies that incorporate lung ultrasound, chest CT, or correlation with inflammatory and radiologic parameters may provide deeper insight into the frequency, mechanism, and clinical implications of pleural effusion in pediatric COVID-19 subtypes.

## Limitations

Despite the strengths of consecutive enrollment and the relatively large sample size, this study has several limitations. Its cross-sectional and single-center design precludes assessment of temporal changes and limits generalizability to other populations.

In addition, not all patients diagnosed with MIS-C had RT-PCR–confirmed SARS-CoV-2 infection; however, all fulfilled the diagnostic criteria of the United States Centers for Disease Control and Prevention (CDC), which allow alternative evidence of current or recent infection, including serology, antigen testing, or documented exposure within the four weeks preceding symptom onset.

Although all measurements were performed by one experienced pediatric cardiologist to minimize inter-observer variability, results remain operator-dependent, and ventricular function was assessed using M-mode–derived LVEF and TAPSE alone, which may lack sensitivity for detecting subtle or regional myocardial dysfunction. Speckle-tracking echocardiography and cardiac MRI were not routinely available, raising the possibility of underestimation of subclinical myocardial or coronary involvement. Formal severity grading of mitral and aortic regurgitation was not performed, and despite applying pathological MR criteria, a proportion of trivial or borderline jets may still represent physiological regurgitation, potentially overestimating valvular involvement. Additionally, absence of multivariable analysis means that residual confounding (e.g., age, ICU admission, variant type, or treatment differences) may influence observed associations, and results should be interpreted cautiously.

Another important limitation of this study is the absence of longitudinal follow-up data. Echocardiographic assessments were performed only during hospitalization, precluding evaluation of the temporal evolution or resolution of cardiac findings, including ventricular dysfunction, valvular regurgitation, and pericardial effusion. Consequently, long-term cardiac outcomes in both MIS-C and non–MIS-C patients could not be assessed.

## Conclusion

In summary, this large echocardiographic study highlights the spectrum of cardiac involvement in children hospitalized with COVID-19, with or without MIS-C. While left ventricular dysfunction and coronary artery abnormalities were relatively uncommon, mitral regurgitation and pericardial effusion were frequently observed during hospitalization, particularly among patients with MIS-C. However, in the absence of a healthy or febrile control group, these findings cannot be considered specific to SARS-CoV-2 infection or MIS-C and may, at least in part, reflect nonspecific responses to acute or critical illness. Pericardial effusion was the only echocardiographic finding statistically associated with ICU admission; nevertheless, no predictive or causal inferences can be drawn from this association given the observational study design.

Importantly, because advanced imaging modalities such as speckle-tracking strain echocardiography and cardiac magnetic resonance imaging were not available, the burden of subclinical myocardial injury may have been underestimated, and our findings primarily reflect abnormalities detectable by conventional echocardiography. Accordingly, the absence of detected myocardial dysfunction should not be interpreted as the absence of myocardial involvement.

These results emphasize the importance of routine echocardiographic evaluation in hospitalized children with COVID-19, especially in the presence of inflammatory features. Future prospective studies with serial imaging and advanced modalities are needed to clarify the natural history and long-term cardiovascular consequences in this population.

## Data Availability

Raw data that support the findings of this study are available from the corresponding author, upon reasonable request.

## Abbreviations

COVID-19: Coronavirus Disease 2019
SARS-CoV-2: Severe Acute Respiratory Syndrome Coronavirus 2
MIS-C: Multisystem Inflammatory Syndrome in Children
ICU: Intensive Care Unit
EF: Ejection Fraction
LV: Left Ventricle
RV: Right Ventricle
TAPSE: Tricuspid Annular Plane Systolic Excursion
MR: Mitral Regurgitation
AR: Aortic Regurgitation
Z-score: Standard Deviation Score
SD: Standard Deviation
CRP: C-Reactive Protein
NT-proBNP: N-terminal pro–B-type Natriuretic Peptide
POCUS: Point-of-Care Ultrasound (if used or implied)
MRI: Magnetic Resonance Imaging
